# Between Life and Death: Sea Urchin Embryos Undergo Peculiar DNA Fragmentation after Exposure to Vanadium, Cadmium, Gadolinium, and Selenium

**DOI:** 10.3390/life14101296

**Published:** 2024-10-12

**Authors:** Chiara Martino, Roberto Chiarelli

**Affiliations:** 1 Department of Biological, Chemical and Pharmaceutical Sciences and Technologies (STEBICEF), University of Palermo, Viale delle Scienze, 90128 Palermo, Italy; roberto.chiarelli@unipa.it; 2 NBFC, National Biodiversity Future Center, Piazza Marina 61, 90133 Palermo, Italy

**Keywords:** apoptosis, DNA fragmentation, metal pollution, marine ecotoxicology, TUNEL assay, sea urchin embryo

## Abstract

Exogenous DNA damage represents one of the most harmful outcomes produced by environmental, physical, or chemical agents. Here, a comparative analysis of DNA fragmentation was carried out on *Paracentrotus lividus* sea urchin embryos exposed to four common pollutants of the marine environment: vanadium, cadmium, gadolinium and selenium. Using the terminal deoxynucleotidyl transferase dUTP nick end labeling (TUNEL) assay, fragmented DNA was quantified and localized in apoptotic cells mapping whole-mount embryos. This is the first study reporting how different chemicals are able to activate distinctive apoptotic features in sea urchin embryos, categorized as follows: (i) cell-selective apoptosis, showing DNA fragmentation restricted to a subset of extremely damaged cells, acting as an embryo survival mechanism; or (ii) total apoptosis, with fragmented DNA widespread throughout the cells of the entire embryo, leading to its death. Also, this is the first report of the effects of Se exposure on *P. lividus* sea urchin embryos. These data confirm the TUNEL assay as the most suitable test to study DNA fragmentation in the sea urchin embryo model system. Taken together, this research highlights embryos’ ability to find alternative pathways and set physiological limits for development under stress conditions.

## 1. Introduction

Chromatin fragmentation is considered a hallmark and marker of irreversible cell death [1]. It is driven by the activation of endonucleases, key enzymes mediating regulated DNA double-strand cleavage, leading to fragments that are multiples of ~185 bp [2]. In physiological conditions, DNA fragmentation represents a controlled and regulated process occurring during specific stages of the cell cycle [3] and is one of the peculiar features among the biochemical events involved in apoptosis [4]. Since apoptosis is crucial for maintaining cell number homeostasis in multicellular organisms, its alteration is often associated with several human pathologies, including cancer [5]. Physiological apoptosis is involved in several key processes, including normal cell turnover, proper functioning of the immune system, embryonic development, and chemically induced cell death [6].

Different chemicals are able to induce cell death via the apoptotic pathway mediated by DNA fragmentation in different systems, from cancer cells [7,8] to marine organisms [9,10]. In the marine ecosystem, metals represent the major cause of chemical pollution from anthropogenic sources, including domestic sewage, industrial effluents, agricultural discharge, mining, coastal construction activities, and dredging [11]. Metals induce severe ecotoxicological problems since they tend to persist in the environment and bioaccumulate in marine animals [12], leading to their biological amplification through the trophic levels in the food chain and ultimately posing severe health hazards to human health [13,14]. Metals can have different negative effects on marine animals, resulting in apoptotic or autophagic induction, antioxidant mechanisms, inflammation, and embryonic abnormalities [15,16].

The sea urchin *Paracentrotus lividus* is a key ecological species in the Mediterranean Sea [17] and its embryos and larvae are extremely sensitive to pollution since they develop in the water column without any maternal protection from environmental stressors, thus representing a useful tool to assess environmental quality [18,19]. When exposed to toxic chemicals, including metals, sea urchin embryos are able to activate a temporal hierarchy of defense strategies, starting with cytoprotection mechanisms such as heat shock proteins (HSPs) and/or metallothionein induction [18,20]. If these mechanisms are not enough to counteract the negative effects of the stressor, then additional strategies are triggered, such as autophagy to remove damaged proteins and organelles and/or apoptosis to remove entire cells [18,21,22,23,24].

In this context, DNA fragmentation thus embodies a pertinent marker of the apoptotic processes induced by chemical agents in sea urchin embryos [25], serving as a crucial cellular signature of the embryonic pathological conditions. Different techniques allow to detect and analyze the specific DNA fragmentation pattern, including the terminal deoxynucleotidyl transferase dUTP nick-end labeling (TUNEL) assay [26], which is considered the most sensitive, accurate and quantitative test [1,4].

Previous studies analyzed the toxic effects and/or potential teratogenic and mutagenic actions of different polluting metals, including vanadium (V), cadmium (Cd), and gadolinium (Gd), on *P. lividus* sea urchin embryos, finding morphological anomalies, abnormal biomineralization, and the activation of the cellular stress response, ultimately leading to autophagy and apoptosis [18,21,22,23,27,28,29,30,31,32,33]. The effects of Se exposure on sea urchin embryos have only been reported for the long-spined sea urchin species *Diadema antillarum* [34], while no data are available on *P. lividus.*

Although these four chemicals are naturally found at doses in the low nM range in the environment due to geological processes, their concentration is rapidly growing in coastal environments due to their increasing use in different industrial and medical processes, since they pass through water treatment facilities unchanged [35,36,37,38,39], reaching a low μM range in seawater around urban areas with large human populations. Specifically, concentrations in polluted seawater around the world reach 120 μg L^−1^ for V [36], 112 pmol kg^−1^ for Gd [40], 12 μg L^−1^ for Cd [41], and 4.74 μg L^−1^ for Se [42], indicating that their pollution is an urgent issue which needs to be addressed.

The aim of this study is to conduct a comparative analysis of DNA fragmentation in *P. lividus* sea urchin embryos exposed to four different pollutants of the marine environment: V, Gd, Cd, and Se. Using the TUNEL assay, it was possible to localize and quantify the fragmented DNA in apoptotic cells, mapping whole-mount embryos. This is the first study reporting how different chemicals are able to activate distinctive apoptotic features in sea urchin embryos, categorized as follows: (i) cell-selective apoptosis, showing DNA fragmentation restricted to a subset of extremely damaged cells, acting as an embryo survival mechanism; or (ii) total apoptosis, with fragmented DNA widespread throughout the cells of the entire embryo, ultimately leading to its death. Also, this is the first report of the effects of Se exposure on *P. lividus* sea urchin embryos.

## 2. Materials and Methods

### 2.1. Embryo Cultures and Chemical Exposure

**Figure 1:**
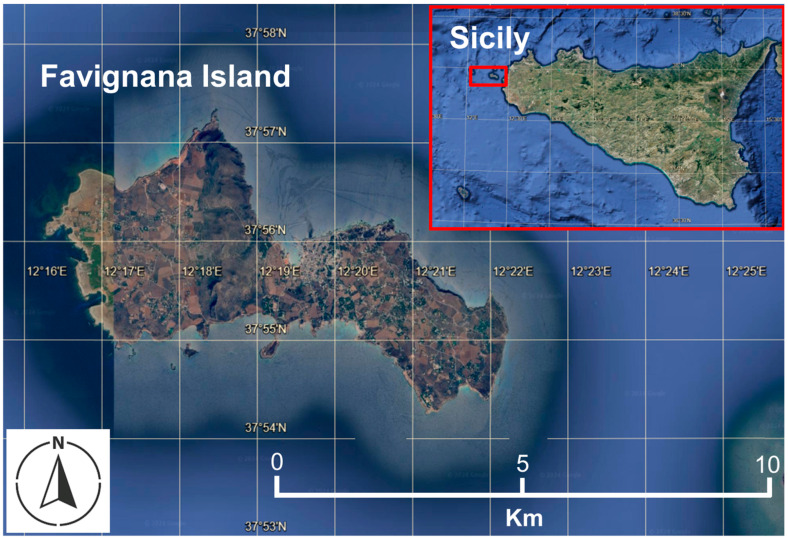
Location of the sampling points for *Paracentrotus lividus* sea urchins in the Favignana island MPA, west coast of Sicily.

Adult specimens of *P. lividus* sea urchins were harvested from the west coast of Sicily, in the Favignana island MPA, Western Mediterranean Sea (coordinates: 37°55′34″ N 12°19′16″ E) (Figure 1), and acclimatized under laboratory conditions at 18 °C until the gamete collection phase.

Three independent experiments were performed, with gametes obtained from at least three males and three females. The gametes were obtained by injection of 0.5 M KCl into the coelomic cavity of adult urchins. Sperm was collected dry, using a Pasteur pipette, and kept on ice, while the eggs were placed in approximately 50 mL of MFSW (Millipore Filtered Sea Water). An aliquot of 10 µL of egg suspension, belonging to each female specimen, was inspected under a light microscope in order to select the eggs according to quality criteria reported elsewhere [24,43,44]. High-quality eggs from multiple females were then mixed, subjected to two washes in MFSW, and suspended in a glass container at a final concentration of 5000 eggs/mL.

An aliquot of 1 µL of dry sperm was diluted in 10 mL MFSW to promote sperm activation, and, subsequently, a morpho-functional analysis was performed using light microscopy. A rapid fertilization test was performed in order to ascertain the complete fertilizing capacity of the gametes, which was 99%. Embryos were developed as described by Chiarelli et al. [19], according to the standard procedures applied in toxicology and in studies about metal exposure in sea urchin embryo cultures [45,46]. Embryos were exposed from fertilization for the following 48 h to 100 μM of the following: sodium orthovanadate (Na_3_VO_4_, hereafter V) (Sigma-Aldrich, St. Louis, MO, USA, cod. S6508); cadmium chloride (CdCl_2_, hereafter Cd) (Sigma-Aldrich, St. Louis, MO, USA, cod. 202908); gadolinium (III) acetate hydrate (Gd(CH_3_CO_2_)_3_·xH_2_O, hereafter Gd) (Sigma-Aldrich, St. Louis, MO, USA, cod. 325678); and sodium selenite (Na_2_SeO_3_, hereafter Se) (Sigma-Aldrich, St. Louis, MO, USA, cod. 214485). This concentration was chosen based on the dose–response results previously obtained for V, Gd, and Cd [19,22,44] and represents the potential environmental risk level.

### 2.2. Larval Morphology

For each experiment, at the pluteus stage (48 h), 100 larvae from each treatment were randomly collected for microscopic examination (Olympus BX50, Olympus Corporation, Tokyo, Japan) and subsequently photographed using a digital camera. The larvae were then scored and categorized on the basis of the observed developmental stage and the detected morphological anomalies. To compare the ability of embryos exposed to the four different chemicals to correctly develop, a common metric of larval growth was measured using ImageJ (ver 1.46 r)—the body length (*n* = 9).

### 2.3. Embryo Fixation and Storage

After 48 h of development/treatment, 5 mL of embryo cultures was recovered and washed twice in MFSW. Fixation of whole embryos was performed in plastic tubes using 4% paraformaldehyde, added in increasing aliquots and incubated for 60 min at room temperature. After two washes in MFSW, fixed embryos were suspended in absolute methanol and stored at −80 °C until use.

### 2.4. TUNEL Assay and Quantitative Fragmented DNA Analysis

An aliquot of embryos suspended in methanol, containing approximately 2500 embryos, was subjected to stepwise rehydration in PBS1X.

The TdT-mediated dUTP nick-end labeling (TUNEL) assay (Promega, Madison, WI, USA, cod. G3250) was performed on whole fixed embryos, as previously described by [24,30], in order to identify nuclei showing DNA fragmentation. Nuclei were observed with a fluorescence microscope (Olympus BX50), using a 20× objective.

Images of the equatorial optical sections of 9 embryos per each treatment were captured by a digital camera (Nikon, Tokyo, Japan) and analyzed using the ImageJ 1.46r software (Bethesda, MD, USA) to quantify the total amount of fragmented DNA, reported as arbitrary units. Data are presented as the mean ± SD (*n* = 9).

### 2.5. Statistical Analysis

Quantitative data related to the BL values and the TdT test were analyzed by a one-way analysis of variance (ANOVA). Tukey’s HSD test was used as a post hoc test for mean comparison. The homogeneity of variance was checked and confirmed using Levene’s test. All statistical analyses were performed using the Statistica 13.2 software (StatSoft, Tulsa, OK, USA), and a *p*-value < 0.05 was considered significant.

## 3. Results

### 3.1. Exposure to Chemical Agents Increases Abnormal Development and Decreases Larval Size

**Figure 2:**
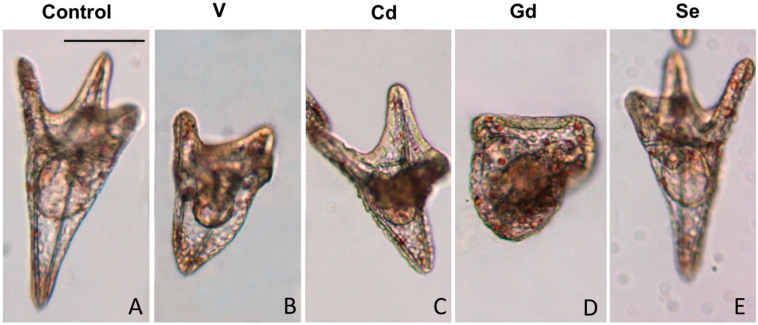
Representative images of 48 h larvae: (**A**) controls; embryos exposed to 100 μM of (**B**) vanadium (V); (**C**) cadmium (Cd); (**D**) gadolinium (Gd); (**E**) selenium (Se). Bar: 100 μm.

**Figure 3:**
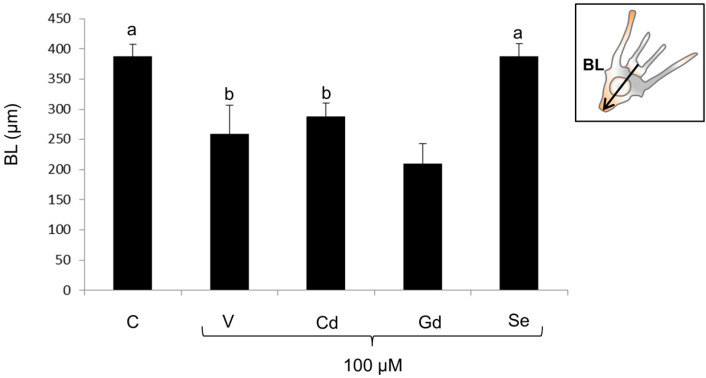
Larval body length (BL) detected after 48 h of development (*n* = 9 ± SD). Treatments with the same letter do not differ (Tukey HSD).

Control larvae grown for 48 h showed a well-developed pluteus morphology with all the organs deriving from the ectoderm, mesoderm, and endoderm being correctly distinguishable (Figure 2A). The controls also displayed an adequate extension of the body due to the presence of a complete and correct skeleton, as shown by the correct size of the BL (388 ± 19.5 μm) (Figure 3).

Except for Se, exposure to chemicals caused developmental delays and anomalies and significantly affected the BL (F_4,40_ = 53.4; *p* < 0.0001). Embryos exposed to V 100 μM showed delays and abnormalities, having an intermediate morphology between the prism and initial pluteus, a shorter skeleton, and a reduction in embryonic mass with a 30% decrease in the BL (Figure 2B and Figure 3). Larvae exposed to Cd 100 μM had an early pluteus morphology, with a shorter skeleton with respect to the controls and, thus, a slight reduction in embryonic mass, featuring a 30% reduction in the BL (Figure 2C and Figure 3). Exposure to Gd 100 μM caused developmental abnormalities and delays, mainly regarding the alteration in skeleton growth in the plutei, causing a peak 46% BL decrease (Figure 2D and Figure 3). Strikingly, exposure to Se 100 μM did not cause any delays or morphological anomalies, as the chemical was well tolerated by the embryos, which presented an architecture (Figure 2E) and a BL (Figure 3) comparable to the controls.

### 3.2. Sea Urchin Embryos Undergo Peculiar DNA Fragmentation after Exposure to Different Chemicals

#### 3.2.1. Control Embryos Show Physiological DNA Fragmentation

**Figure 4:**
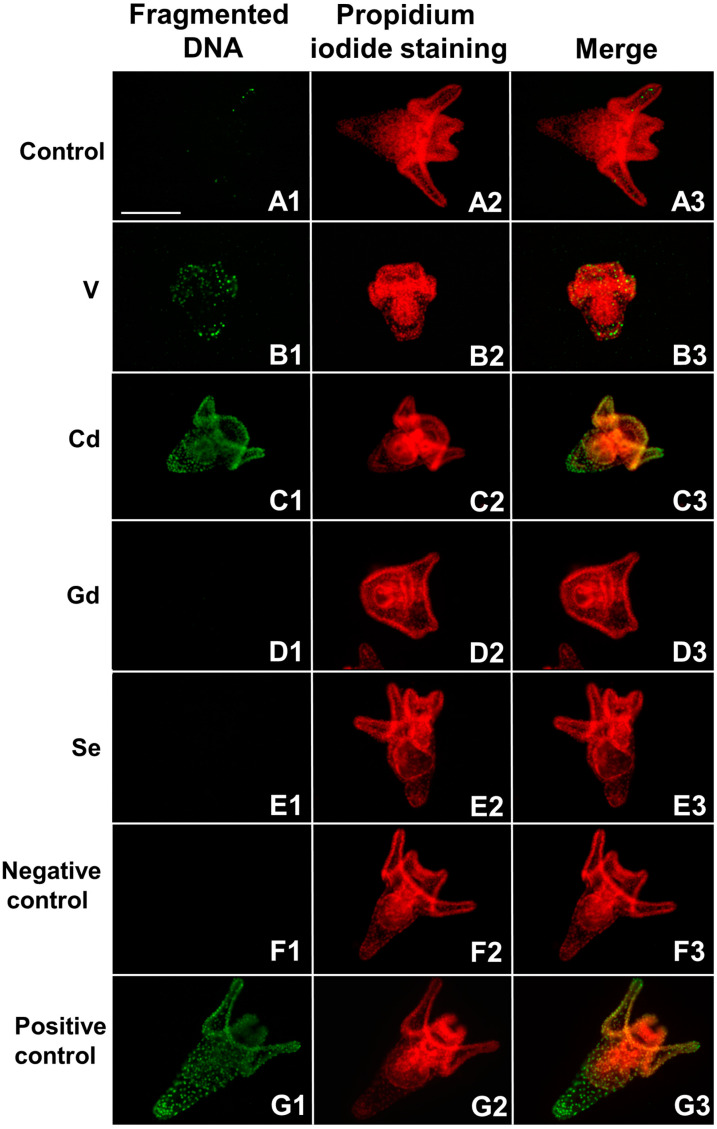
Fluorescent terminal deoxynucleotidyl transferase dUTP nick end labeling (TUNEL) assay on whole-mount sea urchin embryos. Images of representative control and treated embryos at 48 h, observed under a fluorescence microscope: fragmented DNA (**A1**–**G1**); nuclei stained with propidium iodide (**A2**–**G2**); merging of both signals (**A3**–**G3**). Control embryo (**A1**–**A3**); V-treated embryo (**B1**–**B3**); Cd-treated embryo (**C1**–**C3**); Gd-treated embryo (**D1**–**D3**); Se-treated embryo (**E1**–**E3**); negative-control embryo (**F1**–**F3**); and Positive-control embryo (**G1**–**G3**). Bar = 100 μm.

The results obtained by the TUNEL assay in control embryos showed a slight signal related to DNA fragmentation, indicating physiological apoptosis. Specifically, the embryos had a few apoptotic nuclei, mainly located at the level of the post-oral arms, in those tissues destined for cell renewal, since controlled death is a key part of embryonal development, necessary for shaping the body and molding tissues (Figure 4(A1–A3)).

#### 3.2.2. Vanadium: Cell-Selective DNA Fragmentation

The analysis of DNA fragmentation in V-exposed embryos showed cell-selective apoptosis. Specifically, DNA fragmentation was found in a high number of cells at the level of the pre- and post-oral arms and in the mouth region. The fragmented nuclei in these latter tissue districts involved approximately 50% of the cells. A few more cells with fragmented DNA were observed in the apical end region, belonging to cells distributed mainly towards the internal wall of the embryo, and at the level of the intestine (Figure 4(B1–B3)).

#### 3.2.3. Cadmium: Total DNA Fragmentation

Embryos exposed to Cd displayed a total DNA fragmentation affecting, indiscriminately, all nuclei of all tissue districts in the embryos. From qualitative observation, only a gradation of the level of fragmentation was observed, which was greater in the apical end and in the ectoderm cells (Figure 4(C1–C3)).

#### 3.2.4. Gadolinium and Selenium: No DNA Fragmentation

Gd and Se did not induce any DNA fragmentation, and, strikingly, physiological fragmentation was also absent (Figure 4(D1–D3)).

### 3.3. Quantitative and Comparative Analyses of DNA Fragmentation

**Figure 5:**
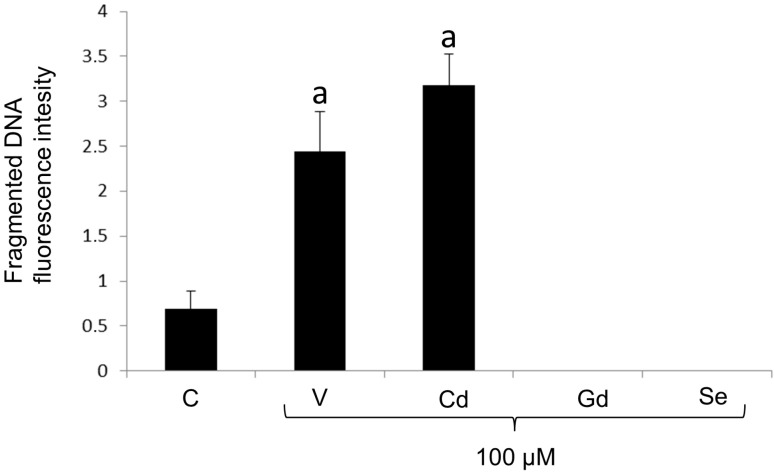
Histogram showing fluorescence optical densitometry analysis related to fragmented DNA signals. Data report the quantification of green fluorescence for the entire morphological population (*n* = 9 ± SD). Treatments with the same letter do not differ (Tukey HSD).

Exposure to the different chemicals had a significant effect on the amount of apoptotic fragmented DNA (F_4,40_ = 40.3; *p* < 0.0001). The results of the quantitative analysis showed that the level of fragmented DNA had a significant increase in embryos exposed to V and Cd, measuring, respectively, 3.5- and 4.6-fold higher than the control, while, in embryos exposed to Gd and Se, no signal correlated with DNA fragmentation was detected (Figure 5).

## 4. Discussion

The conservation of DNA sequence information is a key aspect for the propagation of life. The DNA molecule is highly reactive and prone to chemical modifications by either endogenous or exogenous genotoxic vehicles. In the ecotoxicological context, DNA damage represents one of the most important effects induced by chemical agents [47,48].

DNA fragmentation can be present in marine animals as a consequence of exposure to environmental stressors but can also represents a feature of physiological apoptosis [6].

In sea urchin embryos, it was previously demonstrated the existence of a hierarchical and finely orchestrated response to environmental stressors. All these adaptive responses to environmental changes aim to either buffer the stress or generate alternative developmental phenotypes [49]. This work confirmed that phenotypic plasticity represents the first response to metal pollution, as shown here by the presence of abnormal phenotypes, the decreased ability to reach the larval stage, and the substantial decrease in size in embryos exposed to V, Cd, and Gd. The metal causing the worst developmental abnormalities and the major decreases in larval size, as seen by measuring the BL, was Gd, for which a comprehensive study investigating the effects of a wide range of concentrations on four phylogenetically and geographically distant sea urchin species (including *P. lividus*) had been previously conducted [28]. These changes in size and shape can have harmful consequences on larval fitness, negatively impacting their feeding and swimming abilities [23,50].

In precedent studies of metal contamination by Cd, Gd, and V, it was shown that HSPs are constitutively expressed at low levels and their synthesis is upregulated after stress exposure [18,22]. The autophagic process is then induced to clear cellular vesicles and granules containing the sequestered chemical pollutants or damaged proteins and organelles accumulated if the HSPs’ decontamination mechanism was not enough to offset the threat [21,22,30]. In bivalve mollusks and fishes exposed to changing environmental conditions, it was shown that the repeated activation of autophagy was a key response to generate a significant tolerance to pollutant stressors [51,52].

The apoptotic pathway represents the last line of sea urchin embryos’ defense. In this paper, using the TUNEL assay, a comparative analysis of the DNA fragmentation induced by four chemical agents was carried out, and the results obtained here provide molecular evidence that these different chemicals are able to activate distinctive apoptotic features in sea urchin embryos. This aspect highlights the high sensitivity of sea urchin embryos to specific pollutants and their ability to activate alternative developmental pathways and/or specific defense strategies aimed at developing an adequate molecular and cellular response to selectively respond to the stress.

Specifically, exposure to V activated cell-selective apoptosis, with DNA fragmentation restricted to a subset of extremely injured cells, acting as an embryo survival mechanism since i) nearby cells could profit from the molecules resulting from apoptotic clearance [53] and ii) sacrificing only some cells allowed the embryo to carry on its physiological development program. On the contrary, exposure to Cd induced total apoptosis, with fragmented DNA widespread throughout the cells of the entire embryo, ultimately leading to its death. Since Cd is an extremely toxic and teratogenic heavy metal [38], the defense strategies activated by embryos were not sufficient to buffer the stress and ensure developmental stability; thus, apoptosis was eventually induced in all embryonic tissue districts, leading to death.

Gd and Se exposure did not induce apoptosis. While Se appeared to be well tolerated by embryos, with no evident morphological consequences, previous results using a lower, environmentally relevant Gd concentration (20 μM) demonstrated that exposed embryos activated different defense strategies, from phenotypic plasticity, resulting in alternative developmental phenotypes (altered morphology and skeletogenesis), to altered gene expression and HSPs and autophagy induction to preserve the developmental program, while apoptosis was never activated [22,29]. Strikingly, the concomitant exposure to Gd and increased temperatures (21 and 24 °C) under global warming conditions induced the activation of cell-selective apoptosis [23]. These results highlight the adaptability of embryos’ cellular stress response mechanisms to changing environmental conditions and constitute a warning regarding the embryonic threshold of tolerance when contrasting multiple stressors in a climate change perspective [54].

From an ecological point of view, exposure to toxic chemicals can be an expected problem in development since some toxins occur naturally in the marine environment, such as phytotoxins and microbial byproducts [49]. In a recent paper, exposing *Arbacia lixula* sea urchin embryos to an oxylipins-containing extract obtained from the macroalgae *Ericaria brachycarpa,* it was demonstrated that the dose used was able to move the needle towards cell-selective or total apoptosis [51], highlighting the embryos’ ability to find alternative pathways and set physiological limits for development under stress conditions.

Strikingly, regardless of the stressor (V, Gd and increased temperature, or low doses of the *E. brachycarpa* extract) inducing the cell-selective apoptosis, the involved embryonic districts were roughly the same, indicating that the tissues most damaged and sensitive to environmental stressors coincided. These cells may be replaced later in development.

As shown by the quantitative results, it is worthy to note that exposure to Gd and Se showed no fragmented DNA, indicating that these chemicals were also able to inhibit physiological apoptosis. This aspect represents by itself a harmful alteration in developmental pathways since embryonic programmed cell death is essential for cytodifferentiation and balancing cell proliferation, representing evolutionary conserved mechanisms during development from Nematodes to Echinoderms and Mammals [55,56,57].

## 5. Conclusions

The cellular choice between survival and death in sea urchin embryos is mediated by apoptosis, a highly controlled form of programmed cell death essential for correct embryonic development and maintaining tissue homeostasis. Previous works demonstrated that exposure to pollutants activates the apoptotic program, leading to a cascade of biochemical events, including caspase activation and DNA fragmentation.

The current results denote how apoptosis has the ability to tip the scale in the direction of embryo survival through cell-selective apoptosis or lead to embryo death through total apoptosis. Major concerns arise because the rapid anthropogenic changes to the environment can overpower embryos’ protective strategies. These data also confirm the TUNEL assay as the most suitable test to study DNA fragmentation in the sea urchin embryo model system, since it allows to localize fragmented DNA in apoptotic cells, mapping whole-mount embryos, thus identifying the involved cells and tissues.

## Data Availability

The original contributions presented in this study are included in this article; further inquiries can be directed to the corresponding author.
